# Minimally invasive spinal anesthesia for cesarean section in maternal anticoagulation therapy: a randomized controlled trial

**DOI:** 10.1186/s12871-018-0679-1

**Published:** 2019-01-12

**Authors:** Dan Huang, Linjie Zhu, Jie Chen, Jie Zhou

**Affiliations:** Department of Anesthesiology, Renji Hospital, School of Medicine, Shanghai Jiaotong University, Shanghai, 200127 China

**Keywords:** Minimally invasive, Low back pain, Postdural puncture headache, Spinal anesthesia, Cesarean section, Anticoagulants

## Abstract

**Background:**

Anticoagulant therapy during pregnancy is widely used due to the increasing awareness of maternal hypercoagulability. Few studies have reported the use of minimally invasive spinal anesthesia in these parturients. The objective of this study was to evaluate the safety and feasibility of minimally invasive spinal anesthesia in parturients with anticoagulation therapy undergoing cesarean section.

**Methods:**

This was a randomized, controlled study conducted in 239 parturients using anticoagulants and undergoing selective cesarean section. 37 parturients withdrew, and finally parturients received spinal anesthesia using 27gauge pen type fine spinal needles (experimental group, *n* = 110) and 22gauge traditional spinal needles (control group, *n* = 92). The primary efficacy outcomes included low back pain (LBP) and postdural puncture headache (PDPH) after delivery. Secondary efficacy outcomes included visual analogue scale during subarachnoid puncture (VASdural), difference between visual analogue scale (VAS) during peripheral venipuncture and VASdural (∆VAS), VAS of back puncture point 24, 48 and 72 h after operation (VASdural-24 h, VASdural-48 h and VASdural-72 h, respectively), maternal satisfaction and hospitalization stay.

**Results:**

No parturient had PDPH and was suspected with spinal or intracranial haematoma in two groups. There was no significant difference in VASlbp-24 h, VASlbp-48 h and VASlbp-72 h (*P* = 0.056; *P* = 0.813; *P* = 0.189, respectively) between two groups. In experimental group, VASdural (*P* = 0.017), ∆VAS (*P* = 0.001) and VASdural-24 h (*P* < 0.0001) were lower, whereas maternal satisfaction was higher (*P* = 0.046). There was no significant difference in VASdural-48 h, VASdural-72 h, urination function, strength recovery and hospitalization stay (*P* = 0.069; *P* = 0.667; *P* = 0.105; *P* = 0.133; *P* = 0.754, respectively) between the two groups.

**Conclusions:**

Minimally invasive spinal anesthesia provided lower VASdural, VASdrual-24 h and a higher maternal satisfaction. Hence, it is considered as a safe, reliable and reasonable option for cesarean section parturients during maternal anticoagulation therapy with normal platelet count and coagulation time.

**Trial registration:**

This study was registered at www.ClinicalTrials.gov at November 11th, 2016 (NCT02987192).

## Background

According to the World Health Organization (WHO) Global Survey on Maternal and Perinatal Health in 2010, Chinese health facilities has the highest cesarean section rate (CSR) (46.2%) in Asia (27.3%) [1], and the CSR had risen far in excess of the optimal 15% recommended by WHO [1, 2]. According to a recent study, the overall annual rate of cesarean deliveries between 2008 and 2014 was increased in China, i.e., up to 34.9% [3]. With the implementation of second child policy, the ratio still remained high. Intravertebral anesthesia is the most commonly used method for parturients undergoing cesarean section. Traditional intravertebral anesthesia may cause postdural puncture headache (PDPH) and low back pain.

PDPH may require epidural blood patching, resulting in prolonged hospital stay and emergency visits after discharge [4, 5]. Previous study has shown that the incidence of PDPH was about 7 to 30% [6]. Decrease of intracranial pressure due to cerebrospinal fluid (CSF) leakage from the subarachnoid space is currently the leading cause of PDPH [5]. The PDPH, which causes significant morbidity in obstetric patients, has higher incidence because of the increased CSF pressure related to pregnancy, dehydration, blood loss, postpartum diuresis, hormonal imbalance, high serum estrogen levels, and increased peridural pressure [7].

Low back pain (LBP) commonly occurs during late pregnancy and also after delivery. Several research studies revealed that at least half of the pregnant women is affected by LBP [8, 9]. Persistence of LBP for 6 months after delivery has been reported in 5 to 40% of patients [10]. A retrospective study of 40,057 women demonstrated that cesarean delivery and epidural anesthesia might increase the risk of subsequent chronic LBP [11]. The paraspinal muscular relaxtion with stretching of spinal ligaments and/or localized tissue trauma are accepted as the pathophysiological factors of LBP after puncture.

The concept of minimally invasive spinal anesthesia has been put forwarded due to its appearance of pen type lumbar puncture needle and unceasing enhancement of piercing technology. It refers to puncturing with 27gauge pen type fine needle to reduce the damage of supraspinous, interspinous and yellow ligament by puncture needle.

However, there are relative contraindications to spinal anesthesia, one of which is the use of anticoagulants. Anticoagulant therapy during pregnancy is widely used due to the increasing awareness of maternal hypercoagulability [12]. The pregnant women are forced to accept general anesthesia in order to avoid epidural hematoma and lose the experience of baby birth moment. Minimally invasive spinal anesthesia happens to solve this problem, reducing the side effects without increasing the risk of spinal anesthesia for maternal anticoagulation therapy.

We therefore performed a randomized, controlled trial in parturients undergoing anticoagulation therapy to evaluate the safety and feasibility of minimally invasive spinal anesthesia. We also investigated the use of 27gauge pen type fine spinal needles and 22 gauge traditional spinal needles for spinal anesthesia on PDPH and LBP in parturients with anticoagulation therapy undergoing cesarean section.

## Methods

### Research design

This was a prospective randomized controlled study conducted in accordance with the ethics and data protection of Chinese regulations. The protocol was approved by the ethics committee of Renji hospital, School of Medicine, Shanghai Jiaotong University. Written informed consent was obtained from all the parturients before randomization in this prospective, parallel-arm, randomized clinical trial. This trial was a single-site study conducted at the Shanghai Renji hospital. This study was registered at www.ClinicalTrials.gov (NCT02987192).

### Participants

Consecutive parturients aged ≥18 years with singleton pregnancy puerpera who were using anticoagulation medications and undergoing selective cesarean section were eligible for inclusion between November 2016 and December 2017. Only parturients with the American Society of Anesthesiologists physical status between I and II were included. Exclusion criteria were as follows: parturients with a history of migraine headache, previous history of PDPH, platelet count lower than 70*10^9, International Normalized Ratio (INR) > 1.5, severe cardiopulmonary insufficiency, infection of skin or subcutaneous tissue at the puncture site, lumbar or spinal cord disease, increased intracranial pressure, weight > 100 kg or < 50 kg and height > 165 cm or < 155 cm.

### Randomization

Parturients were randomized in 1:1 ratio to the two study groups by means of a central telephone system to ensure concealed allocation. Randomization was performed using a computer-generated randomization sequence with randomly permuted blocks of 4 or 6. Patient caregivers and investigators collecting the data remained unaware of study-group assignments.

### Interventions

Electrocardiogram, blood pressure, pulse oxygen saturation, and heart rate were measured after the parturients entered the operation room. A 20gauge indwelling needle was placed into the peripheral vein. Thromboelastogram, platelet count and coagulation time were detected. All parturients were hydrated with 8 mL/kg body weight Ringer’s lactated solution before spinal anesthesia. Spinal anesthesia was performed with a standardized technique. All parturients received spinal anesthesia by an experienced anesthetist. The parturients in the experimental group received spinal anesthesia using 27gauge pen type fine needles (HaiSheng, China), and control group parturients received spinal anesthesia using 22gauge traditional needles (HaiSheng, China). Lumbar puncture was performed in the intervertebral space of L2–3 by placing the parturients in the right lateral decubitus position. The parturients were instructed to remain flexed and not to move their heads during the insertion of the needle. Spinal needle was inserted through the longitudinal dural fiber using a midline approach at L2–3. After verifying the free flow of CSF through the needle tip, 0.75% ropivacaine 2 mL was injected within 5 s.

Two consecutive episodes of hypotension (defined as systolic blood pressure less than 80% of baseline) were treated with a “rescue” intravenous bolus of 100μg phenylephrine and volume expansion. When the skin closure begined, intravenous Patient-Controlled Analgesia (PCA) pump was commenced to infuse 100 mL mixture of sufentanil (0.045μg/kg/h), flurbiprofen (45μg/kg/h), and saline at a rate of 2 mL/h using an analgesia pump (Apon, China). PCA administration was continued until 48 h postoperatively in the two groups.

### Outcome measures

The primary efficacy outcomes calculated were LBP and PDPH after puncture. LBP after puncture was defined as the continuous pain and tenderness over the lumbar area around the spinal needle insertion and was recorded using visual analogue scale (VASlbp) on the postoperative days [13]. Pain was defined on a scale of 0 as the absence of pain, and 7–10 as severe pain (disabling; unable to perform daily activities). PDPH was defined according to the International Headache Society Classifications, bifrontal or occipital region headache that remained worse in the upright position and relieved with supine posture and headache within 5 days after dural puncture [14]. Other types of headaches were considered nonspecific and were excluded from the study. The intensity of PDPH was also recorded using visual analogue scale (VASPDPH) on postoperative day 1. Secondary efficacy outcomes included were visual analogue scale during subarachnoid puncture (VASdural), difference between VAS during peripheral venipuncture (VASperipheral) and VASdural (∆VAS), VAS of back puncture point 24, 48 and 72 h after operation (VAS24h, VAS48h and VAS72h), maternal satisfaction and hospitalization days. The safety outcome was the incidence of hypotension after anesthesia in the operation room. The dosage and withdrawal time of anticoagulants were also recorded.

### Sample size calculation

Sample size calculation was based on the data from previous studies. The incidence of PDPH in 22gauge needle was reported as 34% [6] and LBP after caesarean delivery with spinal anesthesia was 31.8% [11], respectively. By assuming a fall by half of the incidences, we calculated that a sample size of 82 parturients per group would be required to achieve a power of 90% with a two-sized α risk of 0.05, which was calculated using PASS software, version 15.0(NSCC, USA).

### Statistical analysis

Categorical data were described as frequencies and proportions and were analyzed using Chi-square test. Kolmogorov-Smirnov test was used to examine the normality of distribution of continuous outcomes. Normally distributed continuous variables were described as mean ± standard deviation (SD) and were analyzed using Student’s *t* test, whereas non-normally distributed continuous variables were presented as median ± interquartile range (IQR) and were performed with the Mann-Whitney U test. Significance was set at 0.05 level. Statistical analyses were performed using GraphPad Prism software, version 6.01(GraphPad Software, Inc. USA).

## Results

### Demographic, physiologic and surgical characteristics

**Fig. 1:**
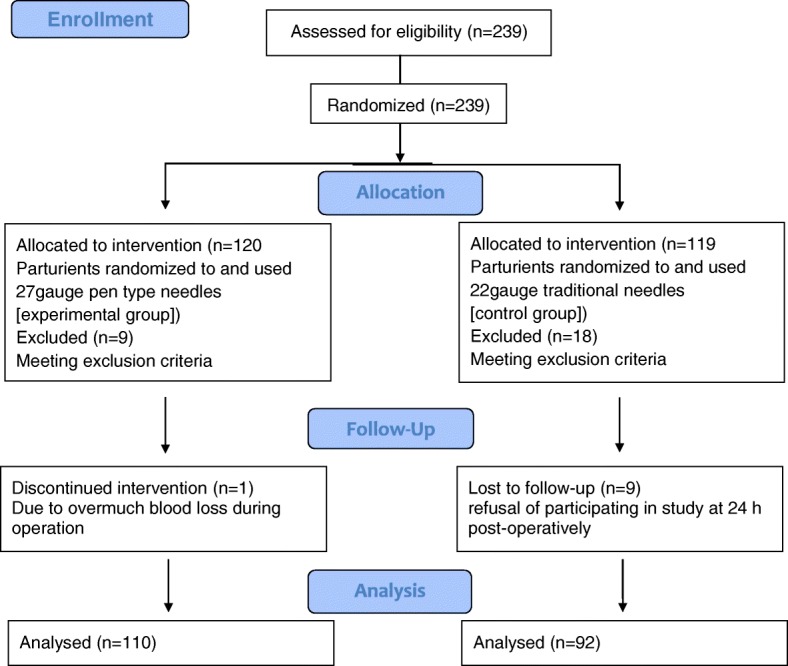
Flow diagram of a trial comparing low back pain, postdural puncture headache, pain of puncture point, maternal satisfaction and hospitalization days between 22 and 27guage needles

A total of 239 parturients were randomly assigned to the experimental group (*n* = 120) or control group (*n* = 119). 37 participants withdrew during our study period; 84.5% of 239 parturients were analyzed in our study (Fig. 1). There were 10 withdraws in the experiment group: 1 subject was transferred to the intensive care unit (ICU) immediately post-operatively because of overmuch blood loss during operation, and other 9 subjects met exclusion criteria. 18 subjects in the control group met exclusion criteria and other 9 subjects decided not to participate in the study further after operation without specific reason. Thus, the final numbers of participants were 110 in experiment group and 92 in control group. Both patient characteristics and surgical characteristics were similar in the two groups. Birthweight was also similar between the groups and Apgar score of each neonate at 1 min and 5 min was 10 (Table 1).

**Table 1:** Baseline Parturients and Surgical Characteristics

Characteristics	Experimental group (*n* = 110)	Control group (*n* = 92)	*P* value
Age, median (IQR), y	32.0(29.0–35.0)	32.0(28.0–35.0)	.474
Height, median (IQR), cm	160.0(158.0–163.0)	161.0(158.0–165.0)	.527
Body mass index, mean(SD)	26.6(3.7)	26.5(3.3)	.869
Gestational weeks, median (IQR)	38.0(37.0–38.0)	38.0(37.0–38.0)	.643
ASA status I, *n* (%)	50(45.0)	38(41.3)	.572
History of spinal anesthesia, *n* (%)	16(14.5)	12(13.0)	.839
Operation time, median (IQR), min	41.0(33.0–50.0)	41.5(35.0–48.0)	.620
Induction-to-delivery time, median (IQR), min	6.0(5.0–10.0)	7.0(6.0–9.0)	.346
Newborn weight, median (IQR), g	3130.0(2830.0–3375.0)	3150.0(2910.0–3410.0)	.538
Hospital stay, median (IQR), d	3.0(3.0–4.0)	3.0(3.0–3.0)	.754

*SD* Standard Deviation, *IQR* Interquartile Range, *ASA* American Society of Anesthesiologists

### Use of anticoagulants and coagulation function

Maternal anticoagulation therapy was shown in Table 2 and Table 3. Daily dosage of low molecular weight heparin (LMWH) was 4100 U. There was no significant difference in the dose and time of anticoagulant between the two groups. Aspirin was the most common anticoagulant medication and alone or duplex anticoagulation medications favored more. There was no significant difference in thromboelastogram, platelet count and coagulation time between the two groups. D-Dimer and α angle in the two groups were higher than normal (Table 4). None of the parturients was suspected of spinal or intracranial hematoma.

**Table 2:** Dosage and Withdrawal Time of Anticoagulant

Variables	Experimental group (*n* = 110)	Control group (*n* = 92)	*P* value
Daily dosage of aspirin, median (IQR), mg	25.0(25.0–50.0)	25.0(25.0–50.0)	.135
Withdrawal time of aspirin, median (IQR), d	4.0(0.3–5)	3.0(1.0–5.0)	.405
Number of aspirin users, n (%)	104(94.5)	88(95.7)	.758
Withdrawal time of LMWH, median (IQR), h	11.0(8.8–13.0)	12.5(11.0–15.0)	.096
Number of LMWH users, *n* (%)	66(60.0)	44(47.8)	.091
Daily dosage of Salvia miltiorrhiza, median (IQR), g	0.3(0.3–0.6)	0.3(0.3–0.6)	.057
Withdrawal time of Salvia miltiorrhiza, median (IQR), d	1.0(0.0–1.0)	0.0(0.0–2.0)	.686
Number of Salvia miltiorrhiza users, *n* (%)	14(12.7)	14(15.2)	.684

*IQR* Interquartile Range, *LMWH* Low Molecular Weight Heparin

**Table 3:** Use of Anticoagulants

Types of drugs	Alone medication	Duplex medication	Triple medication	Total, *n*	%
Aspirin	82	92	18	192	58.2
LMWH	8	84	18	110	33.3
Salvia Miltiorrhiza	2	8	18	28	8.5
Total, *n*	92	92	18		
%	45.5	45.5	8.9		

*LMWH* Low Molecular Weight Heparin

**Table 4:** Thromboelastogram, Platelet count and Coagulation Time

Variables	Experimental group (*n* = 110)	Control group (*n* = 92)	*P* value
D-Dimer, median (IQR)	0.7(0.4–0.9)^a^	0.6(0.4–1.0)^a^	.607
FDP, median (IQR)	5.6(3.5–11.0)	6.9(3.8–9.3)	.627
TT, median (IQR)	16.4(12.9–17.4)	14.5(12.5–16.9)	.055
APTT, median (IQR)	26.7(24.8–28.1)	27.6(25.1–29.0)	.097
PT, median (IQR)	10.7(9.4–11.4)	10.1(9.4–11.5)	.512
Fg, median (IQR)	4.3(3.9–4.9)	4.4(3.9–4.9)	.449
INR, median (IQR)	1.0(0.9–1.0)	0.9(0.9–1.0)	.484
PLT^9, median (IQR)	198.0(150.0–259.0)	194.5(175.0–211.0)	.413
Rtime, median (IQR)	6.6(5.9–7.3)	6.9(6.5–7.4)	.090
Ktime, median (IQR)	1.6(1.4–1.8)	1.6(1.5–1.8)	.053
α angle, median (IQR)	73.5(70.7–75.0)^a^	72.6(71.0–73.6)^a^	.054
MA, median (IQR)	69.4(69.1–70.0)	69.6(69.1–70.0)	.088

^a^D-Dimer and α angle in two groups were higher than normal

*IQR* Interquartile Range, *FDP* Fibrinogen Degradation Product, *TT* Thrombin Time, *APTT* Activated Partial Thromboplastin Time, *PT* Prothrombin Time, *Fg* Fibrinogen, *INR* International Normalized Ratio, *PLT* Platelets, *MA* Maximum Amplitude

### Primary and secondary efficacy outcomes

In the primary efficacy outcomes, no parturient suffered from PDPH and no significant difference in VASlbp-24 h, VASlbp-48 h and VASlbp-72 h between the two groups was observed (*P* = 0.056; *P* = 0.813; *P* = 0.189, respectively). In the secondary efficacy outcomes, VASdural, ∆VAS and VAS24h in the experimental group were lower than control group (VASdural median 3.0 vs 4.0, *P =* 0.017; ∆VAS median 0.0 vs 1.0, *P =* 0.001; VASdural-24 h median 0.0 vs 1.0, *P* < 0.0001). Maternal satisfaction in experimental group was higher than control group (98.2% vs 91.3%, *P* = 0.046). No significant difference in VASdural-48 h, VASdural-72 h and hospitalization stay was observed between the two groups (*P* = 0.069; *P* = 0.667; *P* = 0.754, respectively).

No differences in the feelings during puncture, puncture time, puncture point bleeding, inserting depth of puncture needle and the time of analgesia block level that reached T6 were observed between the two groups (*P* = 0.062; *P* = 0.644; *P* = 0.740; *P* = 0.073, respectively). Parturients in the two groups had similar incidence of hypotension and received similar volume of vasopressor (*P* = 0.123; *P* = 0.073, respectively). There was no significant difference in urination function, strength recovery and remedy for analgesia after operation between the two groups (*P* = 0.105; *P* = 0.133; *P* = 0.344, respectively) (Table 5).

**Table 5:** Primary and Secondary Endpoints

Variables	Experimental group (*n* = 110)	Control group (*n* = 92)	*P*
VASlbp-24 h, median (IQR)	3.0(2.0–4.0)	3.0(3.0–4.0)	.056
VASlbp-48 h, median (IQR)	2.0(1.0–3.0)	2.0(1.0–3.0)	.813
VASlbp-72 h, median (IQR)	1.0(0.0–1.0)	1.0(0.0–1.0)	.189
VASperipheral, median (IQR)	3.0(2.0–5.0)	3.0(3.0–4.0)	.769
VASdural, median (IQR)	3.0(1.0–6.0)	4.0(3.0–6.0)	.017
∆VAS, median (IQR)	0.0(−1.0–1.0)	1.0(0.0–2.0)	.001
VASdural-24 h, median (IQR)	0.0(0.0–1.0)	1.0(1.0–1.0)	<.0001
VASdural-48 h, median (IQR)	0.0(0.0–0.0)	0.0(0.0–1.0)	.069
VASdural-72 h, median (IQR)	0.0(0.0–0.0)	0.0(0.0–0.0)	.667
maternal satisfaction (satisfaction, %)	108(98.2)	84(91.3)	.046
Puncture time, median (IQR), s	40.0(22.0–60.0)	60.0(30.0–68.0)	.062
Puncture point bleeding (bleeding, %)	10(9.1)	11(12.0)	.644
Inserting depth of puncture needle, median (IQR), cm	5.6(5.5–6.0)	6.0(5.3–6.0)	.740
Time of analgesia block level reached T6, median (IQR), min	6.0(6.0–8.0)	8.0(6.0–8.0)	.073
Incidence of hypotension, *n* (%)	60(54.5)	40(43.4)	.123
Rescue phenylephrine required, median (IQR), μg	100.0(100.0–200.0)	100.0(100.0–175.0)	.073
Rescue intraoperative intravenous anesthesia required, *n* (%)	8(7.3)	10(10.9)	.459
Nausea or vomiting, *n* (%)	12(10.9)	8(8.7)	.644
Postoperative self-urination time, median (IQR), h	18.0(15.0–20.0)	19.0(11.0–21.0)	.105
Recovery of lower limb muscle strength, median (IQR), h	4.0(3.0–5.0)	4.0(4.0–6.0)	.133
First remedy for analgesia, median (IQR), h	5.0(2.0–7.0)	4.0(2.8–7.0)	.344

*VASlbp-24 h* Visual Analogue Scale of Low Back Pain 24 h after Operation, *VASlbp-48 h* Visual Analogue Scale of Low Back Pain 48 h after Operation, *VASlbp-72 h* Visual Analogue Scale of Low Back Pain 72 h after Operation, *VASperipheral* VAS during Peripheral Venipuncture, *VASdural* Visual Analogue Scale during Subarachnoid Puncture, *∆VAS* Difference between VASperipheral and VASdural, *VASdural-24 h* VAS of Back Puncture Point 24 h after Operation, *VASdural-48 h* VAS of Back Puncture Point 48 h after Operation, *VASdural-72 h* VAS of Back Puncture Point 72 h after Operation, *IQR* Interquartile Range

## Discussion

Our research indicated that minimally invasive spinal anesthesia was a reliable and reasonable option for cesarean section in maternal anticoagulation therapy with normal platelet count and coagulation time. Previous literature concluded that the anticoagulant treatment continues to be contraindicated to spinal/epidural anesthesia [15]. Spinal-epidural hematoma during spinal anesthesia was described in 1/200000–250,000. Spinal or intracranial hematoma is a rare but a severe complication [16]. Traumatic puncture was assumed to be the risk factor for the development of this kind of hematoma. Anticoagulant treatments increase the risk of spinal hematoma to 1/40000 in patients undergoing anticoagulation treatment [17]. To prevent spinal hematoma, neuraxial anesthesia is prohibited when the anticoagulant treatment is used as curative dose during delivery. These conclusions refer to the relatively coarse technology and traditional lumbar puncture needle. In our study, there was no significant difference in the puncture point bleeding between the two groups. Defecation function, lower limb strength and sensation of all parturients in our study were normal. We inferred that no parturient was suspected of spinal or intracranial hematoma. Minimally invasive spinal anesthesia in our research was considered as safe and effective approach for maternal anticoagulation due to smaller caliber of puncture needle and lesser damage of intraspinal blood vessel.

No parturient had suffered from PDPH and no significant difference in PDPH using 22 or 27gauge needles was revealed in our observation. Smaller gauge needles were recommended for spinal anesthesia to reduce the incidence of PDPH. Kim et al. reported no significant difference in the cause of PDPH by using 23 and 25 gauge needles in spinal anesthesia [18], which was consistent with our study findings. However, our result differed from the conclusion by Lambert et al. that compared with 26gauge Quincke needle, the incidence of PDPH using a 27gauge Quincke needle was significantly reduced [19]. Previous studies have shown that older people have a lower incidence of PDPH than younger people, which may be related to a decrease in pain perception or pain response in the elderly [20, 21]. Age is likely to be an important factor in the pathogenesis of PDPH. However, it was different with our result as our study subjects only included young women subjects. A successful initial lumbar puncture and avoiding local muscle-ligamentous trauma caused by repeated punctures due to the clinician’s technique can be considered the cause of difference.

Our data demonstrated that there was no significant difference in LBP using either 22 or 27gauge needles. Whereas, we found that the procedure of minimally invasive spinal anesthesia provided a lower VAS during subarachnoid puncture, a lower VAS of back puncture point at 24 h after operation and a higher maternal satisfaction. However, the conclusions drawn by previous studies showed discrepancies. Lowery et al. reported that 11% of the oncologic patients were aged between 2 and 17 years, and had LBP after lumbar puncture following the use of a 22gauge cutting Quincke needle [6]. In contrast, Kim M and Yee K, respectively reported no significant difference in pain score in 1–3 days after puncturing with different needles [18, 22]. Our positive result on the advantage of the use of 27gauge puncture needles may be due to the reduction of local muscle-ligamentous trauma associated with needle insertion. It was noteworthy that although effective analgesia, muscular relaxation, immobility, and stressed posture resulted in the postural pain primarily, enormous physical and physiological changes during pregnancy and after delivery such as lumbar lordosis, center of gravity rise and fall, and loss of abdominal muscle support that resulted in intense stretch on the lower back should be also considered [11]. Wang CH [23] concluded that postpartum LBP could be related to changes during pregnancy and not related to spinal anesthesia, and this might be the reason for the negative result from our study in LBP using either 22 or 27gauge needles. A cross-sectional study, which is the largest study on pregnancy-related LBP in the literature, also showed that about 1 in 2 women have pregnancy-related LBP in any stage of pregnancy [9].

The risk of venous thromboembolism (VTE) in pregnancy was 5.7/10000 [24]. Guidelines for anticoagulant indications and management have been published by different national and international agencies to prevent venous thromboembolism during pregnancy [25–27]. Although anticoagulation therapy was given, maternal thrombophilias were still increased among these parturients in our research, some of whom had high D-Dimer, normal platelet count and coagulation time. A therapeutic anticoagulation window of 12 h was recommended during delivery when using LMWH as prophylactic [28]. The mean delay between the last injection and the need for anesthesia in our data was more than 12 h. Aspirin was not recommended for drug withdrawal according to the guidelines. Nevertheless, puerperas in our medical center usually underwent a delay of about 6 days before delivery. The use of anticoagulant therapy during pregnancy should be cautious because of the potential for both fetal and maternal complications.

Furthermore, our study showed that aspirin was the most common anticoagulant medication used by pregnant population in our medical center. Aspirin is a preferred anticoagulant and is widely accepted by doctors and parturients in our medical center, occupying the largest proportion of usage. It is suggested that oral low-dose aspirin treatment can prevent the occurrence of preeclampsia, but there are complication of placental infarction [29]. Currently, based on epidemiological evidence, it is recommended to take low-dose aspirin daily as a preventive intervention for high-risk preeclampsia during pregnancy after 12 weeks of gestation [30]. The preference of aspirin usage in our medical center may be due to the prevention of fetal growth restriction and a reversible increase in uterine blood flow [31], whereas evidence on the use of LMWH from randomized control trials in both in vitro and in vivo studies remained inconsistent [32–36]. The use of heparin, including LMWH, was established for the prevention/improvement of thrombophlebitis in the pathological hypercoagulability during pregnancy [37]. Meanwhile, the dosage regimen of coagulant used in our medical center mainly focused on alone and duplex medications rather than triple combination. In women with antiphospholipid (aPL) syndrome with recurrent miscarriages, to improve blood circulation, guidelines recommend an anticoagulant therapy of oral low-dose aspirin combined with intravenous LMWH [37, 38]. Further prospective randomized controlled trials are required to determine the optimal maternal hypercoagulant treatment strategy. Although the beneficial effects of Salvia Miltiorrhiza on anticoagulation, vasodilation, reduction of oxidative stress, and lipid profiles have been put forwarded, studies on Salvia Miltiorrhiza in obstetrics were limited [39]. Morton JS et al. investigated the use of Salvia Miltiorrhiza did not affect placenta- related factors and blood pressure of pregnant women with pregnancy-induced hypertension [40]. Hence, further study on larger sample size is required.

## Limitation of the study

Insufficient sample size was the main limitation of our research. Our study did not include twins and obese parturients due to their distinctive body sizes. Because of the difficulty in studying the aim of the study in a prospective way and ethical limits, no magnetic resonance imaging (MRI) was performed on any parturient to check spinal or intracranial hematoma. In addition, since PDPH and LBP were developed till 5 to 7 days after dural puncture, the restricted observation period due to short hospital stay and lack of long-term follow-up information remained as limitations. Besides, our study was a single center study and requires recruitment of more parturients, and monitors for longer follow-up period to obtain confirmatory conclusion and explore the cause and mechanism in future study.

## Conclusion

In conclusion, the present study demonstrates that minimally invasive spinal anesthesia provided a lower VAS during subarachnoid puncture, a lower VAS of back puncture point 24 h after operation and a higher maternal satisfaction. Hence, it was considered to be a safe, reliable and reasonable approach for parturients undergoing cesarean section in maternal anticoagulation therapy with normal platelet count and coagulation time. As the date of delivery is difficult to predict, minimally invasive spinal anesthesia could be a potential solution for parturients taking anticoagulants to go through cesarean section successfully by avoiding general anesthesia and get the experience of joy of baby birth moment.

## Abbreviations

aPL: Antiphospholipid
CSF: Cerebrospinal fluid
CSR: Cesarean section rate
ICU: Intensive care unit
INR: International normalized ratio
IQR: Interquartile range
LBP: Low back pain
LMWH: Low molecular weight heparin
MRI: Magnetic resonance imaging
PDPH: Postdural puncture headache
SD: Standard deviation
VASdural: Visual analogue scale during subarachnoid puncture
VASdural-24 h: Visual analogue scale of back puncture point 24 h after operation
VASdural-48 h: Visual analogue scale of back puncture point 48 h after operation
VASdural-72 h: Visual analogue scale of back puncture point 72 h after operation
VASlbp: Visual analogue scale of low back pain
VASPDPH: Visual analogue scale of postdural puncture headache
VASperipheral: Visual analogue scale of during peripheral venipuncture
VASlbp-24 h: Visual analogue scale of low back pain 24 h after operation
VASlbp-48 h: Visual analogue scale of low back pain 48 h after operation
VASlbp-72 h: Visual analogue scale of low back pain 72 h after operation
VTE Venous: Thromboembolism
WHO: World health organization
∆VAS: Difference between visual analogue scale during peripheral venipuncture and visual analogue scale during subarachnoid puncture
